# The Role of ASIC1a in Inflammatory Immune Diseases: A Potential Therapeutic Target

**DOI:** 10.3389/fphar.2022.942209

**Published:** 2022-07-08

**Authors:** Yinghong Wang, Xiaojie Hu, Yancai Sun, Yan Huang

**Affiliations:** ^1^ Department of Pharmacy, Anhui Provincial Cancer Hospital, The First Affiliated Hospital of USTC, Division of Life Sciences and Medicine, University of Science and Technology of China, Hefei, China; ^2^ Anhui Provincial Laboratory of Inflammatory and Immunity Disease, Anhui Institute of Innovative Drugs, School of Pharmacy, Anhui Medical University, Hefei, China

**Keywords:** ASIC1a, inflammatory, immune, diseases, target

## Abstract

It is acknowledged that chronic inflammation is associated with a rise in extracellular proton concentrations. The acid-sensing ion channel 1a (ASIC1a) belongs to the extracellular H^+^-activated cation channel family. Recently, many studies have been conducted on ASIC1a and inflammatory immune diseases. Here, in this review, we will focus on the role of ASIC1a in several inflammatory immune diseases so as to provide new perspectives for clinical treatment.

## Introduction

**FIGURE 1:**
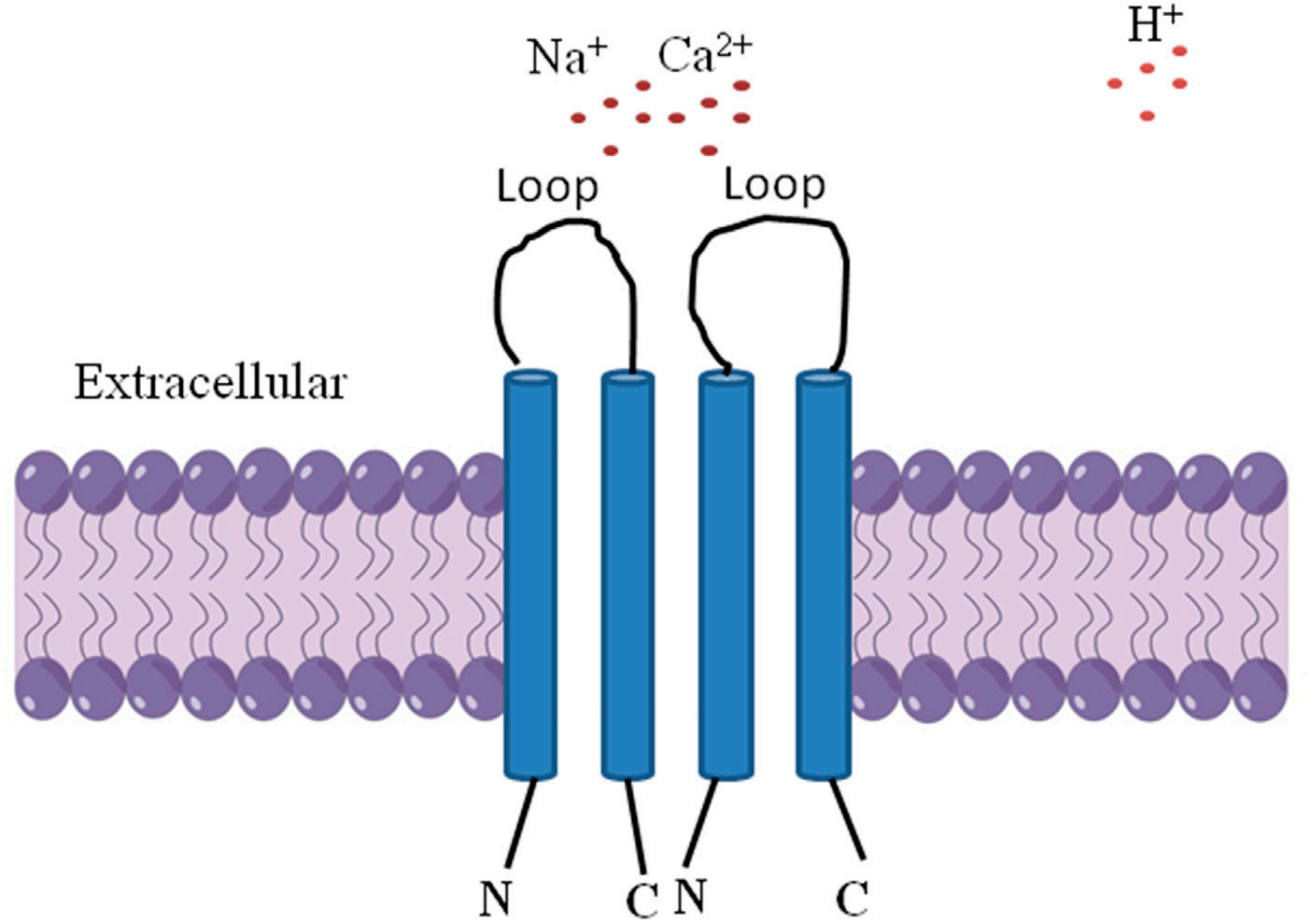
The structure of ASIC1a is comprised of three identical 1a subunits, intracellular amino terminal and carboxyl terminal, two membrane spaning domains and an extracellular loop, which can be activated by extracellular H^+^, open channel has permeability to Na^+^, Ca^2+^.

Acid-sensing ion channels (ASICs) are a subfamily of degenerin/epithelial Na^+^ channel family of non-voltage gated cation channels, currently classified as ligand-gated ion channels, including ASIC1, ASIC2, ASIC3, and ASIC4, which are widely expressed in both the peripheral and central nervous systems. ASICs regulation may occur through various means: multitudinous classes of drugs including small molecules, individual chemical entities, protein–protein interactions including toxins and venoms, metals, signal transduction pathways including receptor–receptor initiated activity, and even endogenous compounds (Alvarez de la Rosa et al., 2000; Zha et al., 2006; Zeng et al., 2014; Wang et al., 2016). Numerous studies have illustrated that ASICs are directly activated by extracellular protons, are shown to contribute to a variety of pathophysiological condition that involves tissue acidosis (Ziemann et al., 2008; Wang and Xu, 2011; Wang et al., 2016; Salinas Castellanos et al., 2021; Xu et al., 2022). ASIC1a is a kind of cation channel protein complex made up of intracellular amino terminal and carboxyl terminal, two membrane spaning domains and an extracellular loop, which can be activated by extracellular H^+^, open channel has permeability to Na^+^,Ca^2+^ (Figure 1) (Zha et al., 2006; Baconguis et al., 2014; Yoder et al., 2018; Zhu et al., 2020; Wang et al., 2021).

Recently there is increasing evidence that ASIC1a plays an important role in the occurrence and development of diseases involving the central and peripheral nervous systems (Zhang et al., 2020; Kong et al., 2021; Qi et al., 2022). It is acknowledged that chronic inflammation is associated with a rise in extracellular proton concentrations. Recently there is considerable evidence that extracellular acidification is a common phenomenon that is relevant to the physiological and pathological mechanism of inflammation, including infectious diseases, early wound healing and tumorigenesis et al. (Riemann et al., 2015; Riemann et al., 2019). The drop in pH occurs during inflammation as a result of the infiltration of inflammatory cells into the tissue, which causes the tissue to burn more energy and oxygen as well as increase its glucose consumption *via* glycolysis and thus increased lactic acid secretion (Xu and Chen, 2020a). Meanwhile, researches show that ASIC1a plays a key role in infiltration and activation of inflammatory cells, which has been detected in different kinds of inflammatory cells, including macrophages, dendritic cells, central microglia, Th cells, eosinophils, neutrophils, mast cells, B cells, plasma cells, and T cells (Tong et al., 2011; Yu et al., 2015; Ni et al., 2018; Tang et al., 2021). In addition, studies also have shown that inflammatory cytokines IL-1β, IL-6, and TNF-α are also closely associated with ASIC1a, which can affect the expression of ASIC1a (Zhou et al., 2015; Zhou et al., 2018). These inflammatory cells involved in ASIC1a are highlighted in Table 1.

**TABLE 1:** The inflammatory cells involved in ASIC1a.

Inflammatory cells type	Expression and major physiological function	References
Macrophages	Bone marrow derived macrophages express ASIC1, and ASIC1 contribute to increased endocytosis of FITC-dextran and upregulated expression of CD80, CD86, and MHC-II in BMMs stimulated by acidosis	Kong et al. (2013)
Microglia	ASIC1 in microglia is increased upon stimulation with LPS. Blocking of ASIC1a reduced the expression of inflammatory cytokines	Yu et al. (2015)
Dendritic cells	Dendritic cells express ASICs, acidosis upregulated the expression of CD11c, MHC class II, CD80, and CD86 and enhanced the Ag-presenting ability of dendritic cells *via* ASICs	Tong et al. (2011)
T cells	T cells show ASIC1 expression at the protein level, ASIC1 has no significant role in T cell function in EAE	Friese et al. (2007)
B cells	Asic1 mRNA is detected in B cells	Friese et al. (2007)
Eosinophils		
Neutrophils	ASIC1a is detected in these five inflammatory cells, indicating the key roles of ASIC1a in the infiltration and activation of these inflammatory cells	Tang et al. (2021)
Mast cells		
Plasma cells		
Th cells		

It is also remarkable that inflammation and trauma can lead to enhanced pain sensitivity, which is closely related to ASICs. Research suggested that ASICs in dorsal horn neurons are involved in central sensory transmission and modulation and may play a role in inflammation-related persistent pain (Wu et al., 2004). In a later study, Ca^2+^ signaling in spinal dorsal horn neurons is enhanced following peripheral inflammation *via* upregulation of ASIC1a channels, which contributes to the hypersensitivity of the dorsal horn neurons and inflammatory processes. Blocking Ca^2+^ permeable ASIC1a channels can result in antinociceptive effects by minimizing or preventing the development of central sensitization induced by inflammation pain (Duan et al., 2007). Further evidence has shown that hyperalgesia is associated with increased mRNA expression of ASIC1a, 1b, and three in the dorsal root ganglions (DRGs) on the ipsilateral side (Nagae et al., 2006; Fu et al., 2016). In this review, we mainly focus on ASIC1a involved in inflammatory immune diseases, in order to provide new perspectives for clinical treatment.

## The Central Nervous System

It is acknowledged that microglia are the most important innate immune cells in the brain and play a vital role in CNS homeostasis, which act as agents of surveillance and scavengers of immune defense and inflammatory response. Yu et al. (2015) identified the existence of ASICs in cultured and *in situ* rat microglia. They found the expression of ASIC1 and ASIC2a in microglia are increased upon stimulation with LPS. Furthermore, ASIC1a blocker amiloride and PcTx1 reduced the expression of inflammatory cytokines, including inducible nitric oxide synthase and cyclooxygenase 2 activated by LPS. Overall, their results suggest that ASICs are involved in the neuroinflammatory response.

Here, we focus on two common central nervous system diseases and review the relationship with ASIC1a to develop a novel therapeutic approach for the control of neuronal diseases related to inflammation.

### Parkinson’s Disease

Parkinson’s Disease (PD), the second-most common neuro-degenerative disease, is characterized by the progressive and selective destruction of dopaminergic neurons located in the substantia nigra pars compacta (SNpc) area of the brain and cytoplasmic inclusions of alpha-synuclein (Mango and Nisticò, 2021; Zhang et al., 2021). Presently available treatments for PD are limited, mostly symptomatic, and associated with decreasing effectiveness and unwanted side effects over time. Treatment strategies targeting the underlying pathogenesis of the disease in order to slow down or halt disease progression, together with reliable and sensitive tests for early detection of the disease, which represents a large unmet medical need (Gelders et al., 2018). The involvement of the innate and adaptive immune systems in neurodegeneration has been documented by a number of postmortem, brain imaging, epidemiological, and animal studies (Chen et al., 2005; Theodore et al., 2008; Sanchez-Guajardo et al., 2010; Harms et al., 2018). Multiple studies have indicated that there were elevated levels of proinflammatory interleukins IL1β, IL2, IL6, TNF-α, and TGFβ1 in the striatum, in addition, concentrations of TNF-α, IL1β, IFNγ, NOS, and ROS were also found to be increased in the substantia nigra of postmortem samples (Mogi et al., 1994; Mogi et al., 1995; Mogi et al., 1996). Furthermore, these findings support the idea that microglia initiate both pro- and anti-inflammatory events, pointing towards that there are multiple phenotypes and distinct roles in PD. As for the association between PD and ASIC1a, Arias et al. (2008) established a model of PD accompanying lactic acidosis by MPTP, they explored the effects of amiloride in the MPTP-treated mouse, and found that amiloride can protect substantia nigra neurons from MPTP-induced degeneration. Moreover, amiloride also preserved dopaminergic cell bodies in the SNc. Parkin encodes a 465 amino acid protein that is expressed in multiple tissues as an E3 Ub-ligase within the ubiquitin (Ub) system, mutations in the parkin gene cause an autosomal recessive form of Parkinson’s disease that develops at a young age (Shimura et al., 2000). Joch et al. (2007) found by knocking out parkin, hippocampal neurons of parkin knockout mice exhibit prominent potentiation of native ASIC currents that are normally suppressed by endogenous parkin in wild-type neurons, which indicated that ASIC1a may be crucially involved in the pathophysiology of PD. Even so, the role of ASIC1a on PD remains unclear.

### Multiple Sclerosis

Multiple Sclerosis (MS) is a chronic autoimmune inflammatory disease which can lead to motor deficit by neuronal damage-induced inflammation and demyelination in the central nervous system. Generally, it is considered to be an autoimmune and T cell-mediated disease. Chronic neuropathic pain is the most serious symptom which severely reduces quality of life (Friese et al., 2014). Roles of ASIC1a in MS were explored by many studies. The experimental autoimmune encephalomyelitis (EAE) model is a well-accepted animal model of MS (Friese et al., 2007; Bjelobaba et al., 2018). To explore the effect of ASIC1 on MS, Friese et al. (2007) established EAE model and ASIC1^−/−^ mice were used, results showed clinical defificit and axonal degeneration markedly reduced compared to wild-type mice. Moreover, pH measurements in the spinal cord of EAE mice indicated tissue acidosis could lead to ASIC1 open. Therefore, they consider that ASIC1 blockers could provide neuroprotection in MS. Vergo et al. (2011) found that ASIC1 was upregulated in axons and oligodendrocytes within lesions from mice with acute EAE and from patients with active multiple sclerosis. Furthermore, ASIC1 co-localization with the axonal injury marker beta amyloid precursor protein indicated ASIC1 was closely related to axonal damage, meanwhile, they found there was a significant decrease in disease severity in amiloride-treated EAE mice compared with vehicle-treated EAE mice, blocking ASIC1 with amiloride protected both myelin and neurons from damage in the acute model. They also examined the role of ASIC1 expression in the oligodendrocytes, results showed in longitudinal white matter tissue sections of acute EAE, there was a significantly increased number of ASIC1a mRNA positive and ASIC1^+ve^ oligodendrocytes in EAE. Taken together, their findings indicate that blockade of ASIC1 has the potential to provide both neuro- and myelo-protective benefits in MS. Similarly, Arun et al. (2013) also found ASIC1 was increased in axons and oligodendrocytes in chronic inactive lesions of cases with progressive MS, axons with an injury profile were continually seen to co-express ASIC1, while ASIC1-positive oligodendrocytes were also identified in chronic MS lesions, indicating a molecular signature that may contribute to cellular damage. And they recruited a group of patients with primary progressive MS participating in a longitudinal imaging protocol to assess whether amiloride could impact on surrogate imaging markers of neurodegeneration, results showed there was statistically significant MRI evidence of benefit during the amiloride phase in progressive MS using small numbers of patients. Their results further extend evidence of the role of ASIC1 to neurodegeneration in MS and indicate that amiloride may exert neuro-protective effects in patients with progressive MS. As for mechanisms of ASIC1a involved neurodegeneration and axonal dysfunction in MS, it is acknowledged that Ca^2+^ plays an important role in this process. Friese et al. (2014) summarized that ASIC1 promotes further Ca^2+^ influx and potentiates Na^+^ influx. While reduced ATP levels lower Na^+^/K^+^-ATPase activity, leading to reverse operation of NCX and increasing intracellular Ca^2+^ and Na^+^ levels. Eventually Ca^2+^ overload activates degradative enzymes and NOS which can lead to neuronal apoptosis and necrosis.

It is recognized that inflammation is characterized by edema, redness, fever, and pain (Kim et al., 2018; Reis et al., 2019). The inflammatory process of the CNS is accompanied by cerebral edema. Recent researches show Piroxicam may play a neuroprotective role in cerebral ischemia by inhibiting aquaporin 4 (AQP4) and ASIC1a, which may indicate a potential association between AQP4 and ASIC1a (Bhattacharya et al., 2012; Mazumder and Borah, 2015). Here, we also briefly introduce AQP4 in order to provide new understanding about the mechanism of ASIC1a in the process of CNS inflammatory. Aquaporins (AQPs) are plasma membrane channels that aid in the bidirectional transmembrane transport of water, which is an important component of cytotoxic edema. AQP4 is the main member of this family expressed in the CNS, an abundance of which is found in astrocytes, which appears to play different roles in CNS edema development and resolution. Therefore, reversible inhibition of AQP4 function during the acute phase may help prevent CNS edema (Kitchen et al., 2020; Liu et al., 2021; Sylvain et al., 2021). The results of previous studies on a single representative water channel protein suggest narrow channels conduct water, whereas wider channels permit solutes to pass through. Kitchen et al. found that single amino acid substitutions in the selectivity filters of AQP1, AQP4, and AQP3 differentially affect glycerol and urea permeability in an AQP-specific manner. Their data showed that substrate discrimination in water channels relies on a complex interplay between the solute, pore size, and polarity, and that using single water channel proteins as representative models has led to an underestimation of this complexity (Hara-Chikuma and Verkman, 2006; Kitchen et al., 2019). The role of water homeostasis in buffering the ion concentration which is mediated mainly by glial cells through the function of water channels. When inflammation occurs, both AQP4 and ASIC1a may be open at the same time, whether there is a certain association between AQP4 and ASIC1a in inflammatory diseases of CNS, relative research should be studied in the future. Targeting the subcellular relocalization mechanisms of aquaporins and ASIC1a will provide an alternative strategy for developing drugs. The definition of signature motifs and molecular mechanisms of ion transport also will provide new avenues for future drug discovery (Markou et al., 2022; Salman et al., 2022; Wagner et al., 2022).

### Rheumatoid Arthritis

Rheumatoid arthritis (RA) is a chronic autoimmune disease characterized by synovial cell proliferation, multiple inflammatory cell infiltration, pannus formation, cartilage and bone tissue destruction, which eventually results in joint deformity and loss of function (Gibofsky, 2014). Recently, it has been reported that ASIC1a was closely related to RA, numerous studies have been conducted on ASIC1a and RA. To explore the roles of IL-1β and TNF-α in acid-induced apoptosis of chondrocytes, Zhou et al. (2018) studied rat adjuvant arthritis and primary articular chondrocytes as *in vivo* and *in vitro* models, they found that IL-1β and TNF-α simultaneously increased ASIC1a expression in articular chondrocytes on a time- and dose-dependent basis. Their results indicated that these cytokines activated MAPK and NF-κB pathways in chondrocytes, and inhibitors of these pathways suppressed the ASIC1a upregulation caused by IL-1β and TNF-α. Furthermore, results showed IL-1β and TNF-α induced ASIC1a promoter activity in chondrocytes by increasing binding to DNA by NF-κB. Additionally, IL-1β and TNF-α decreased cell viability while enhanced LDH release, intracellular Ca^2+^ concentration elevation, loss of mitochondrial membrane potential, cleaved PARP and cleaved caspase-3/9 expression, and apoptosis in acid-stimulated chondrocytes. Their results indicated that IL-1β and TNF-α can enhance acidosis-induced cytotoxicity through increasing the expression of ASIC1a in primary articular chondrocytes. Pyroptosis is a type of proinflammatory programmed cell death that involves the activation of caspase-1 and the production of interleukin IL-1β/18. There has been previous evidence that pyroptosis may be related to the development of some autoimmune diseases, such as RA (Choulaki et al., 2015). Wu et al. (2019) found extracellular acidosis significantly increased the expression of ASIC1a, IL-1β, IL-18, ASC, NLRP3, and caspase-1, while PcTX1 inhibited these effects. Simultaneously, in rats with AA, there were higher levels of the proinflammatory cytokines IL-1β and IL-18 than in rats with normal blood, but aspirin and amiloride suppressed these effects. According to these results, they concluded that ASIC1a is required for pyroptosis to occur in chondrocytes from AA rats, which may be related to ASIC1a’s ability to activate Ca^2+^ inflow, drugs targeting pyroptosis and IL-1β/18 might be developed that can effectively treat RA in the future. Moreover, to identify the role of ASIC1a, calpain, calcineurin, and NLRP3 inflammasome proteins in regulating acid-induced articular chondrocyte pyroptosis, in Zu’s research, chondrocytes from primary rat articular articular cartilage were exposed to different pH, different time, and different treatments including or without ASIC1a, calpain-2, and calcineurin, respectively. They found that extracellular acidosis increased the protein expression of ASIC1a in a pH- and time-dependent manner, and the messenger RNA and protein levels of calpain, calcineurin, NLRP3, and apoptosis-associated speck-like protein were also increased in a time-dependent manner. Interestingly, they also indicated that inhibiting ASIC1a, calpain-2, or calcineurin could decrease the cell death. As a result, they concluded that extracellular acidosis activated ASIC1a, causing pyroptosis of chondrocytes in rat articular cartilage, mechanisms for which may be partly linked to calpain-2/calcineurin activation (Zu et al., 2020). In addition, ASIC1a is also involved in synovial invasion. Niu et al. (2020) investigated the roles of ASIC1a in synovial invasion *in vivo* as well as the migration and invasion of RA-FLS *in vitro*. They found that ASIC1a was highly expressed in RA synovial tissues and RA-FLSs, ASIC1a inhibition by PcTX-1 reduced synovial invasion and expression of MMPs2, 9, and p-FAK to prevent cartilage degradation in AA rats. Furthermore, they also showed that ASIC1a-RNAi and PcTX-1 inhibited the acidity-induced invasion and migration of RA-FLSs while overexpression of ASIC1a increased their expression of MMP2, MMP9, and p-FAK. And in this process Ca^2+^ influx through ASIC1a activated the Ras-related calcium chelating agent Rab1 (Rac1), which was decreased by BAPTA-AM, an intracellular calcium chelating agent. Similarly, Rac1 specific blocker NSC23766 reduced migration and invasion of RA-FLSs as well as MMP2, MMP9 and p-FAK expressions. As a result of their study, they concluded that ASIC1a may be a master regulator of synovial invasion *via* the Ca^2+^/Rac1 pathway. Meanwhile, Zhang et al. (2020) indicated that ASIC1a induces synovial inflammation, which leads to the progression of RA, it was found that ASIC1a was significantly expressed in RA synovial tissues and in human primary RASF as well as in the ankle synovium of AA rats in their research. They showed that activation or overexpression of ASIC1a in RASF enhanced inflammatory cytokines RANTES, sTNF RI, MIP-1a, IL-8, sTNF RII, and ICAM-1, with RANTES increasing most prominently. Furthermore, *in vivo* they found activation of ASIC1a also led to inflammation, synovial hyperplasia, articular cartilage destruction, and bone destruction, resulting in the progression of AA. In summary, these findings offer a rationale for ASIC1a as a potential therapeutic target for RA.

### Asthma

Asthma is a chronic obstructive disease of the airways worldwide, characterized by airway inflammation and airway hyperreactivity (AHR) (Alwarith et al., 2020; Aghapour et al., 2022). Treatment of asthma is a complicated problem, current drug treatments mainly include long-term control treatments, such as inhaled corticosteroids, long-acting beta-agonists, and oral medications. The active search for new therapeutic drugs and methods is concerned by scholars (Chen et al., 2015; Vasileiadis et al., 2019). The study of ASIC1a in asthma is now being slowly recognized. Faisy et al. (2007) found the pH-induced relaxation of airway basal tone was inhibited by ASIC1a inhibitor and the initial pH-induced airway relaxation may be independent of sensory nerves, indicating a regulation of airway basal tone mediated by ASICs on smooth muscle. Reznikov et al. (2016) found ASIC1a protein in vagal ganglia neurons and they induced AHR by sensitizing mice to ovalbumin, results showed that ASIC1a^−/−^ mice did not exhibit AHR while accompanied by a strong inflammatory response. They assessed inflammation using quantitative histopathology and found that OVA-sensitization increased bronchovascular inflammation in both wild-type and ASIC1a^−/−^ mice but there was no difference between two genotypes, therefore they concluded that loss of ASIC1a decreases AHR without alleviating the inflammatory response. Interestingly, they found that IL-13 was increased in the bronchoal veolar lavage fluid of OVA-sensitized ASIC1a^−/−^ mice, but the underlying mechanism of IL-13 upregulation is uncertain, it is considered that ASIC1a deficiency may prevent proton-mediated repression of IL-13 release and/or transcription. Moreover, in this project, levels of substance P was decreased in OVA-sensitized ASIC1a^−/−^ mice bronchoalveolar lavage fluid, which is acknowledged to contribute AHR. Collectively, their results suggest that ASIC1a plays an important role in AHR and can support the possibility that inhibiting ASIC1a might be beneficial in asthma.

### Chronic Rhinosinusitis

Chronic rhinosinusitis with nasal polyps (CRSwNP) is a chronic inflammatory disease, characterized by nasal congestion, rhinorrhea, and diminished smell, as well as headache (Nguyen et al., 2020). On histologic examination, CRSwNP exhibits infiltration of numerous inflammatory cells within the sinuses. A complex interaction between inflammatory cells and mediators plays an integral role in the pathogenesis of CRSwNP (Terna et al., 2016; Schleimer, 2017). Tang et al. (2021) focused on the pathogenesis of CRSwNP and the role of ASIC1a. First, they measured pH values in nasal secretions from control subjects and CRSwNP patients with and without asthma, as well as ASIC1a expression and co-localization in inflammatory cells located in nasal tissue samples from CRSwNP subjects. Then within dispersed nasal polyp cells’ (DNPCs) cultures *in vitro*, they quantified ASIC1a, LDH activity, HIF-1α, and inflammatory cytokines levels. Results showed there were lower pH values and increased levels of ASIC1a protein and mRNA in CRSwNP with asthma, ASIC1a protein was also found in various types of inflammatory cells, while amiloride can reverse this effect. Based on these results, they considered that ASIC1a upregulation may play a key role in sensing acidification and triggering inflammatory responses by enhancing HIF-1α expression and LDH activity, which involved activating inflammatory cells in the pathogenesis of CRSwNP, especially in CRSwNP with asthma.

## Discussion

ASIC1a desensitization causes a dilemma for neurophysiologists studying ASICs, after rapid desensitization induced by acidification, how can ASIC1a function properly in chronic inflammation? Xu et al. indicated that desensitization of the ASIC1a channel by moderate pH decreases suppressed subsequent activation of the ASIC1a current, but do not prevent acid-induced neuronal death. Their results demonstrated that ASIC1a channels play a vital role in mediating neuronal necroptosis in response to extracellular acidification, however, they discovered the ion-conducting mechanisms of the ASIC1a channels do not seem to be essential in this process. Although it cannot be excluded that ionic fluxes *via* the channel pore may play a modulatory role, it seems that RIP1 activation and not ionic conduction per se is essential for acid-induced necroptosis (Wang et al., 2015). Furthermore, in subsequent studies, they showed that the N-terminus (NT) of ASIC1a interacts with its CT to form an auto-inhibition that prevents RIPK1 recruitment/activation under resting conditions. This interaction is disrupted by acidosis by the involvement of glutamate residues at the distal NT. In the presence of mutant ASIC1a containing glutamate-to-alanine substitutions at distal NT, constitutive cell death occurs. N-ethylmaleimide-sensitive fusion ATPase (NSF) further disrupts NT-CT interactions, which corelated with ASIC1a-NT under acidosis, facilitating RIPK1 interaction with ASIC1a-CT (Wang et al., 2020). The results by Xu et al. may partly explain the mechanism after desensitization. On the other hand, it is recognized that plasma membrane expression is critical to the function of ASICs, which act as extracellular proton sensors. In part, the number and function of receptors on the plasma membrane depend on protein synthesis and degradation processes as well as dynamic trafficking processes (Waldmann and Lazdunski, 1998; Zeng et al., 2014; Xu and Chen, 2020b). Therefore, we consider that increased ASIC1a expression and membrane transport can occur after desensitization, which may maintain subsequent function of ASIC1a in inflammatory. Of course, further research is necessary to support this claim. In this review, we focused on inflammatory immune diseases including PD, MS, RA, asthma, and chronic rhinosinusitis, summarized the effects and related mechanisms of ASIC1a, however, there are still few studies of ASIC1a in inflammatory immune diseases, we need to pay more attention in the future.

## Conclusion

Recent studies have shown that ASIC1a plays a vital role in the occurrence and development of diseases involved in the central and peripheral nervous system. In this review, we concluded that ASIC1a is closely associated with inflammation and immunity, which may be a promising target. A deeper understanding of the role of ASIC1a in inflammatory immune diseases will present the potential of using ASIC1a as a new targeting agent for inflammatory conditions. In addition, in the future, for potential targets identified in research, high-throughput screening and computer-aided drug design can be used for novel drug discovery in the field of pharmaceutics, which can provide a novel insight and support target validation in future studies (Aldewachi et al., 2021; Salman et al., 2021).

## Data Availability

The raw data supporting the conclusion of this article will be made available by the authors, without undue reservation.
